# Improving Biomedical Science Literacy and Patient-Directed Knowledge of Tuberculosis (TB): A Cross-Sectional Infodemiology Study Examining Readability of Patient-Facing TB Information

**DOI:** 10.3389/bjbs.2024.13566

**Published:** 2024-10-22

**Authors:** Caoimhe Shannon, Beverley C. Millar, John E. Moore

**Affiliations:** ^1^ School of Biomedical Sciences, Ulster University, Coleraine, United Kingdom; ^2^ Laboratory for Disinfection and Pathogen Elimination Studies, Northern Ireland Public Health Laboratory, Belfast City Hospital, Belfast, United Kingdom

**Keywords:** health literacy, patient education, patient-centred care, tuberculosis, TB, readability

## Abstract

**Background:**

Tuberculosis (TB) continues be the leading cause of death globally due to an infectious agent. There is a paucity of data describing the readability of patient-facing TB information for service users. The aim of this study was to calculate the readability of multiple global TB information sources.

**Methods:**

Information on tuberculosis (n = 150 sources) included nine categories, *Patient-facing information:* WHO publications (n = 17), International governments (n = 19), Hospitals (n = 10), Non-government organisations (NGOs)/charities (n = 20), Cochrane Plain Language Summaries (n = 20); LabTestsOnlineUK (n = 4) and *Scientific-facing information:* Clinical trials (n = 20), Cochrane abstracts (n = 20), Scientific abstracts (n = 20). Readability was calculated using Readable software, defined by (i) Flesch Reading Ease (FRE), (ii) Flesch-Kincaid Grade Level (FKGL), (iii) Gunning Fog Index and (iv) SMOG Index and two text metrics [words/sentence, syllables/word].

**Results:**

Mean readability values for TB information for the FRE and FKGL were 35.6 ± 1.6 (standard error of mean (SEM)) (US Target ≥60; UK Target ≥90) and 12.3 ± 0.3 (US Target ≤8; UK Target ≤6), respectively, with mean words per sentence and syllables per word of 17.2 and 1.8, respectively. Cochrane Plain Language Summaries had similar readability scores to their matching scientific abstract (p = 0.15). LabTestsOnlineUK yielded a mean FRE score of 51.5 ± 1.2, a mean FKGL score of 10.2 ± 0.5 and text metric scores of 16.7 ± 2.3 and 1.6, for words per sentence and syllables per word, respectively. In descending order, TB information from international governments, hospitals and LabTestsOnlineUK were the most readable (FRE = 57.9, 54.1 and 51.5, respectively), whereas scientific abstracts and Cochrane abstracts were the most difficult to read (13.0 and 30.2, respectively).

**Conclusion:**

Patient-facing TB information analysed had poor readability. Effective communication of biomedical science concepts and information relating to TB is vital for service users to enhance their health literacy of tuberculosis, thereby promoting better clinical outcomes. Biomedical scientists are important custodians of scientific information for their service user populations, including other healthcare professionals within the TB multidisciplinary (MDT) team and patient service users. When preparing TB information, this should be checked and modified in real time employing readability calculators, to align with health readability targets.

## Introduction

Historically, tuberculosis (TB) has been the leading cause of death globally due to an infectious agent [1]. A total of 1.3 million people died from TB in 2022 (including 167,000 people with HIV), with an estimated 10.6 million people developing TB worldwide, including 5.8 million men, 3.5 million women and 1.3 million children [1]. Furthermore, it is estimated that more than 1 billion people have succumbed to the disease over the past two centuries [2, 3]. With the occurrence of the COVID-19 pandemic, TB was replaced with SARS-CoV-2, as the leading cause of death globally due to an infectious agent, however with the reduction in deaths due to COVID-19, TB has now regained its position as the leading cause of death from an infectious agent [4]. TB predominantly affects several regions around the world, where most cases are seen in Africa (23%), South-East Asia (46%) and the Western Pacific (18%) (World Health Organisation, 2023) [4]. For the year 2021, the World Health Organization listed the following eight countries as high-incidence locations, including; Bangladesh, China, India, Indonesia, Nigeria, Pakistan, Philippines, and South Africa [4] TB is also present in developed Western nations, including UK, Ireland, USA and Australia [4].

Drug-resistant *Mycobacterium tuberculosis* adds to the global burden of the disease [5]. Multidrug-resistant tuberculosis (MDR-TB) is defined as TB disease that has developed resistance to first-line anti-TB drugs, including isoniazid and rifampicin [5]. Two factors promote drug-resistant TB, extrinsic factors such as social determinants of health in combination with control and prevention services, and intrinsic factors including genetic mutations [6]. Social determinants of health including social, and economic circumstances, impact both TB health and epidemiology. Studies conducted have confirmed that the following factors increase the probability of an individual contracting TB, including living or working in a high-incidence area, low-income, overcrowding, and malnutrition [7–9]. Additionally, inadequate education and access to healthcare results in non-compliance to treatment regimens and thus reduced treatment outcomes [9]. To further support this theory, a study conducted by Mekonnen and Azagew [10] confirmed that non-adherence to anti-tuberculosis treatments, led to the development of increased drug resistance. Consequently, the World Health Organization created the “*End TB Strategy”* in 2015, aiming to achieve a world free from the TB epidemic, with zero suffering and deaths. The strategy developed, focused on guiding global efforts towards the disease, as well as providing access to care and prevention [11].

Research has shown that limited health literacy has an existing relationship with negative health consequences, including the spread of diseases such as TB and MDR-TB [12]. Individuals need adequate literacy skills in order to understand and navigate healthcare systems, preventing the spread of disease. Education has the potential to improve an individual’s literacy skills, however, individuals learn in various ways and basic literacy skills do not guarantee understanding, particularly within the scientific and medical field [13]. Furthermore, hard-to-reach populations, and populations where drug treatments are used frequently, often have low health literacy levels, thus reinforcing little to no knowledge and awareness with regards to TB onset, diagnosis, and treatment regimens [14].

Establishing the quality of a written piece of text and its level of readability ease is a crucial factor to health literacy [15]. Health education materials frequently ignore individuals’ literacy and comprehension skills, resulting in insignificant information being conveyed. Individuals suffering from TB or MDR-TB, encounter several complex decisions regarding treatment that require an in-depth understanding of the potential risks and benefits involved [16]. For example, patients need to decide whether to complete the lengthy 6-month treatment regime which could potentially result in side effects including loss of appetite, and fatigue [17]. However, factors such as low literacy skills and language barriers, affect this decision-making ability. Patient education resources have the potential to assist in the general public’s understanding and insight into TB and MDR-TB, however such materials must be readable, accessible, and tailored to the target audience. Recently in February 2024, the National Institute for Health and Care Excellence (NICE) published an updated guideline on tuberculosis, which covers preventing, identifying and managing latent and active tuberculosis (TB) in children, young people and adults [18]. In this (*1.1.2 Providing information for the public about TB*), specific guidance is given regarding the preparation of educational information on TB, which should be tailored to the target population’s needs and be simple and succinct [18].

The hypothesis of the current study is that freely accessible TB information is not presented in a format that is easily readable by the general population, including service users, patients, families, carers and friends. To investigate this hypothesis, the study had the following objectives including, (i) to identify and examine the readability of public/patient-facing TB information (n = 150 sources), (ii) to establish how readable public-facing TB information is compared to UK and US health-literacy readability reference standards and (iii) to compare readability scores across nine different information sources on TB, including (i) the World Health Organization (WHO), (ii) international governments, (iii) hospitals, (iv) non-government organisations (NGOs)/charities, (v) clinical trials, (vi) Cochrane plain language summaries, (vii) matching Cochrane abstracts and (viii) scientific abstracts and (ix) LabTestsOnlineUK.

## Methods

**FIGURE 1:**
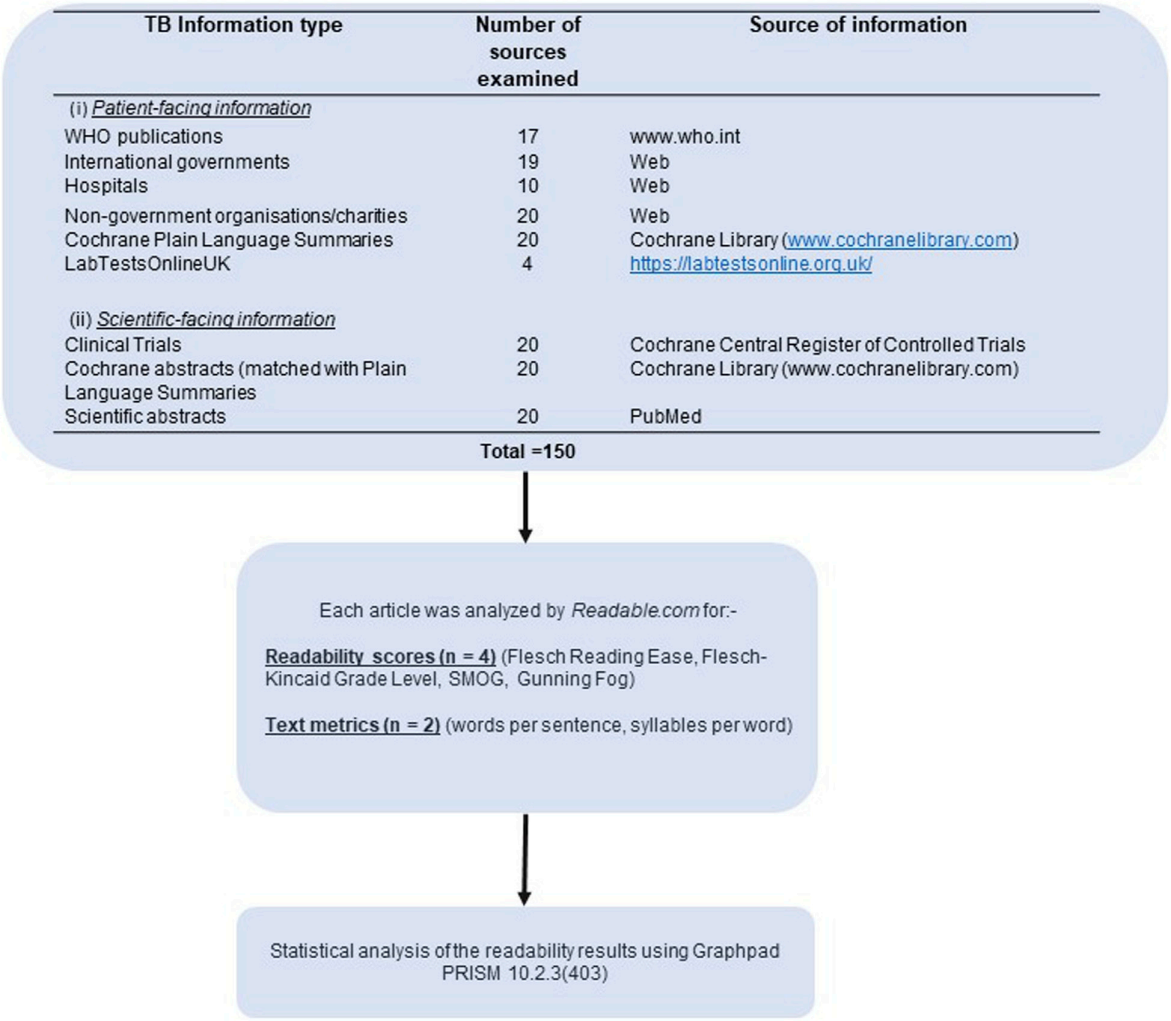
Flow diagram of methodological investigations undertaken in this study and sources of Tuberculosis information.

An overview of the methods employed is shown in Figure 1.

### Retrieval of Information on Tuberculosis

Information on tuberculosis (n = 150 sources) was obtained from publicly and freely available TB web resources, as detailed in Figure 1. The 150 sources of information were composed of nine categories, including:- Patient-facing information: WHO publications (n = 17), International governments (n = 19), Hospitals (n = 10), Non-government organisations (NGOs)/charities (n = 20), Cochrane Plain Language Summaries (n = 20); LabTestsOnlineUK (n = 4): Scientific-facing information: Clinical trials (n = 20), Cochrane abstracts (n = 20), Scientific abstracts (n = 20).

### Determination of Readability Scores and Text Metrics

Each source of TB information was processed from its text, PDF or URL source, using the online subscription-based software, *Readable* (https://www.readable.com), which was used as guided by the manufacturer. All readability analyses were performed on text written in the English language. Readability values, including the Flesch Reading Ease, Flesch-Kinkaid Grade Level, Gunning Fog Index and SMOG Index were calculated, as detailed in Table 1 [15]. An additional two text metrics were also calculated, including words/sentence and syllables/word, as is generally the case with such studies [19]. http://Readable.com was the software of choice as it has been used in several readability and recent studies within healthcare [19]. McGrath and colleagues concluded that *Readable* was the best analytical tool to employ in readability studies, due to its user experience, capacity and accuracy [19].

**TABLE 1:** Readability formulae employed in this study.

Readability formulae	
Flesch Reading Ease (FRE)	$206.835-1.015\;\left(\frac{total\;words}{total\;sentences}\right)-84.6\;\left(\frac{total\;syllables}{total\;words}\right)$
Flesch Kincaid Grade Level (FKGL)	$0.39\;\left(\frac{total\;words}{total\;sentences}\right)+11.8\;\left(\frac{total\;syllables}{total\;words}\right)-15.59$
The Gunning Fog Index	$0.4\times \left[\left(\frac{total\;words}{total\;sentences}\right)+100\;\left(\frac{complex\;words}{total\;words}\right)\right]$
The SMOG Index	$3+\sqrt{polysyllabic\;}count$

The Gunning Fog index was used to assess how clear and straightforward the analysed publications were, determining if the general public could easily comprehend the information provided. Additionally, the SMOG index was used due to its popularity within the healthcare sector.

Complex words are those containing three or more syllables, e.g., tuberculosis, *Mycobacterium*.

Polysyllabic words are words with multiple syllables (i.e., >1 syllable).

### Readability of Infection-Related Non-tuberculosis Information (Control)

Infection-related but not tuberculosis scientific abstracts (n = 20) were examined for their readability, to serve as a control and comparator of the tuberculosis information. These consisted of COVID-19 and other infection–related information [20–39].

### Statistical Analyses

The readability data obtained underwent statistical analyses using GraphPad PRISM version 10.2.3 (403) (Boston, United States). To determine if the data followed a normal distribution, a normality test was performed on each set of data using the Shapiro-Wilk Test. Dependent on the normality of data distribution, for data that were normally distributed, one-way ANOVA (parametric) was performed to compare the means of normally distributed parameters. Data sets that were not normally distributed, the Kruskal-Wallis (non-parametric) test with Dunn’s Adjusted p values was performed. For pairwise comparisons with normal distribution, an unpaired t-test was performed. For those pairs with a non-normal distribution, an unpaired Mann-Whitney test was applied. A p-value of <0.05 (5%) was considered as statistically significant.

## Results

**FIGURE 2:**
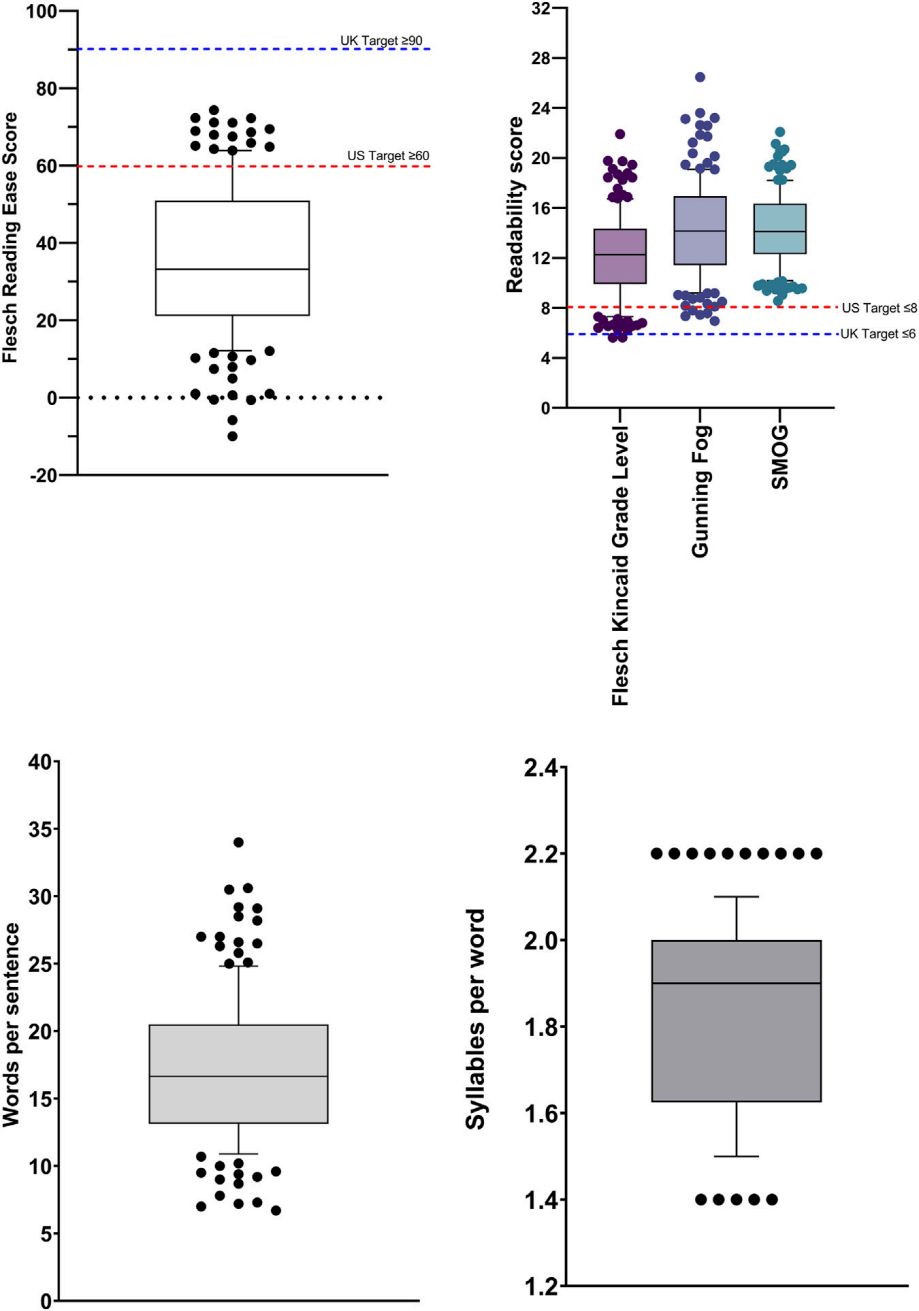
Box and whisker plot comparing readability scores calculated from tuberculosis information sources (n = 150). Flesch Reading Ease; Flesch-Kincaid Grade Level; Gunning Fog Score; SMOG score; Words per sentence; Syllables per word. Box represents 25th and 75th percentile and bar represents the median. Whiskers represent the 10th and 90th percentile and ● represent outliers outside these percentile ranges. The dashed red line represents the target readability score for US readers and the blue line, UK readers.

**FIGURE 3:**
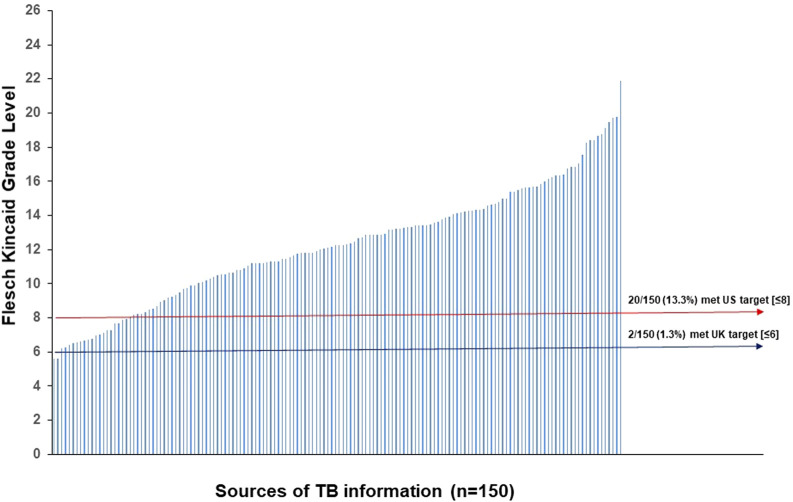
Flesch Kincaid Grade Level readability scores for all 150 sources of information. The red line represents the target readability score for US readers and the blue line, UK readers.

**FIGURE 4:**
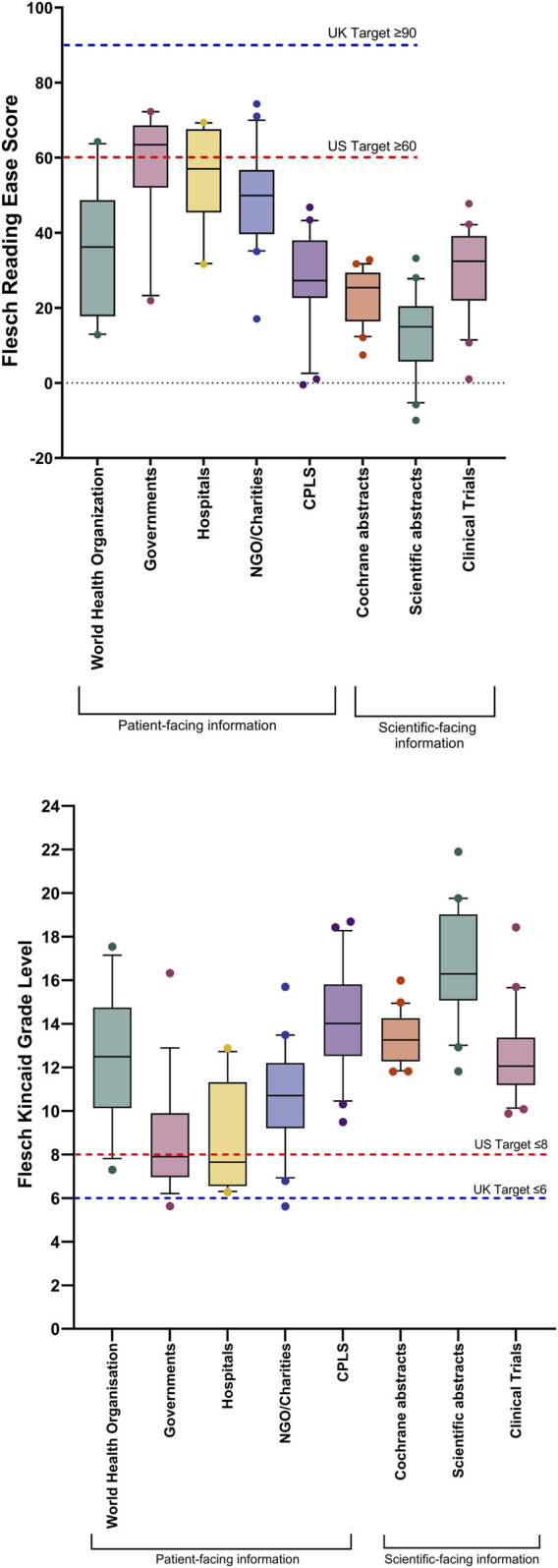
Box and whisker plot comparing readability scores comparing eight categories of tuberculosis information, divided into patient-facing information and scientific-facing information: Flesch Reading Ease; Flesch-Kincaid Grade Level. Box represents 25th and 75th percentile and bar represents the median. Whiskers represent the 10th and 90th percentile and ● represent outliers outside these percentile ranges. Statistical significance is shown, calculated using the Kruskal-Wallis (non-parametric) test with Dunn’s Adjusted p values. A p-value of <0.05 (5%) was considered as statistically significant. The dashed red line represents the target readability score for US readers and the blue line, UK readers. For statistical significance for Flesch Reading Ease between categories, please see Table 2. For statistical significance for Flesch Kincaid Grade Level between categories, please see Table 3. CPLS = Cochrane Plain Language Summaries, Patient-facing information: WHO publications (n = 17), International governments (n = 19), Hospitals (n = 10), Non-government organisations (NGOs)/charities (n = 20), Cochrane Plain Language Summaries (n = 20). Scientific-facing information: Clinical trials (n = 20), Cochrane abstracts (n = 20), Scientific abstracts (n = 20).

**FIGURE 5:**
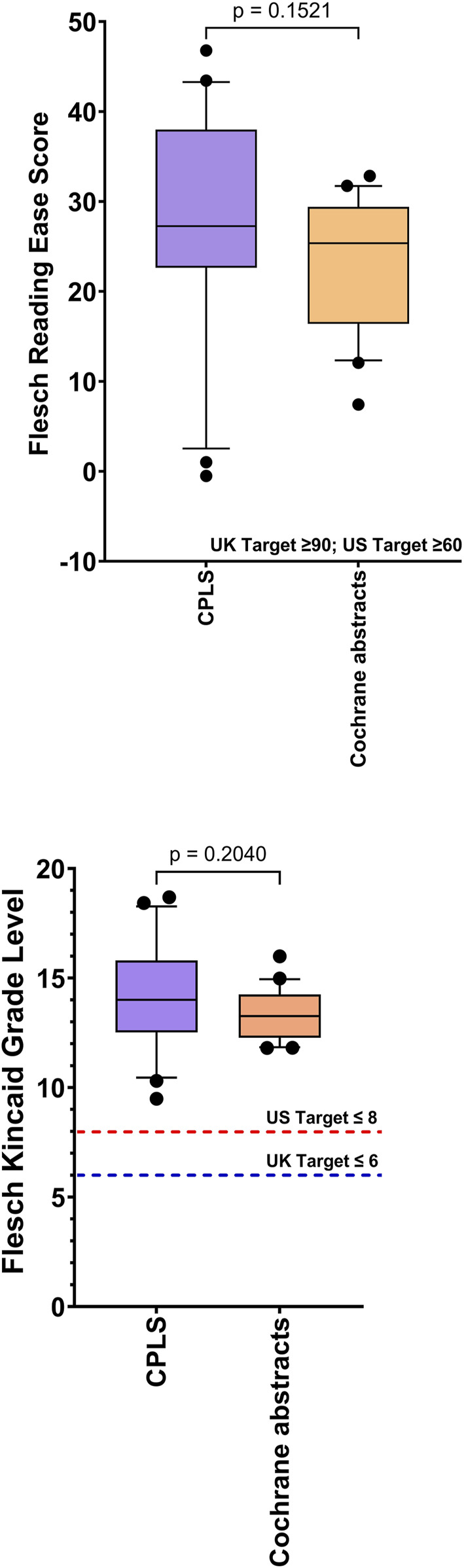
Comparison of the readability of Cochrane Plain Language Summaries (n = 20) v matched Cochrane Scientific Abstracts (n = 20): Flesch Reading Ease; Flesch Kincaid Grade Level Box represents 25th and 75th percentile and bar represents the median. Whiskers represent the 10th and 90th percentile and ● represent outliers outside these percentile ranges. Statistical non-significance is shown, calculated using a parametric unpaired t-test. A p-value of <0.05 (5%) was considered as statistically significant. CPLS = Cochrane Plain Language Summaries.

Readability scores for all 150 combined TB information sources, for the Flesch Reading Ease, the Flesch-Kincaid Grade Level, the Gunning Fog score and the SMOG score, are shown in Figure 2. Mean values for the Flesch Reading Ease and Flesch Kincaid Grade Level were 35.6 ± 1.6 (standard error of mean (SEM)) (US Target ≥60; UK Target ≥90) and 12.3 ± 0.3 (US Target ≤8; UK Target ≤6), respectively, with mean words per sentence and syllables per word of 17.2 and 1.8, respectively. Text metrics scores are shown in Figure 2, respectively. Figure 3 shows the Flesch Kincaid Grade Level readability scores for all 150 sources of information, showing the proportion of information sources examined meeting the UK and US targets for this readability parameter. Comparison of FRE and FKGL readability scores for the eight categories of the TB information are shown in Figure 4 and in Tables 2, 3. Comparison of the readability of Cochrane Plain Language Summaries and their matching scientific abstracts are shown in Figure 5.

**TABLE 2:** Statistical significance for Flesch Reading Ease between tuberculous information categories.

World Health Organisation vs. Governments	0.0402
World Health Organisation vs. Scientific abstracts	0.0088
Governments vs. CPLS*	0.0004
Governments vs. Cochrane abstracts	<0.0001
Governments vs. Scientific abstracts	<0.0001
Governments vs. Clinical Trials	0.0021
Hospitals vs. CPLS*	0.0198
Hospitals vs. Cochrane abstracts	0.0004
Hospitals vs. Scientific abstracts	<0.0001
NGO/Charities vs. CPLS*	0.0183
NGO/Charities vs. Cochrane abstracts	0.0002
NGO/Charities vs. Scientific abstracts	<0.0001

Statistical significance was calculated using the Kruskal-Wallis (non-parametric) test with Dunn’s Adjusted p values. A p value of <0.05 (5%) was considered as statistically significant.

CPLS, Cochrane Plain Language Summaries.

NGO, Non-governmental organisation.

**TABLE 3:** Statistical significance for Flesch Kincaid Grade Level between tuberculous information categories.

World Health Organisation vs. Governments	0.0285
World Health Organisation vs. Scientific abstracts	0.0142
Governments vs. CPLS	<0.0001
Governments vs. Cochrane abstracts	0.0002
Governments vs. Scientific abstracts	<0.0001
Governments vs. Clinical Trials	0.0287
Hospitals vs. CPLS	0.0006
Hospitals vs. Cochrane abstracts	0.0052
Hospitals vs. Scientific abstracts	<0.0001
NGO/Charities vs. CPLS	0.0097
NGO/Charities vs. Scientific abstracts	<0.0001
Scientific abstracts vs. Clinical Trials	0.0046

Statistical significance was calculated using the Kruskal-Wallis (non-parametric) test with Dunn’s Adjusted p values. A p value of <0.05 (5%) was considered as statistically significant.

CPLS, Cochrane Plain Language Summaries.

NGO, Non-governmental organisation.

**FIGURE 6:**
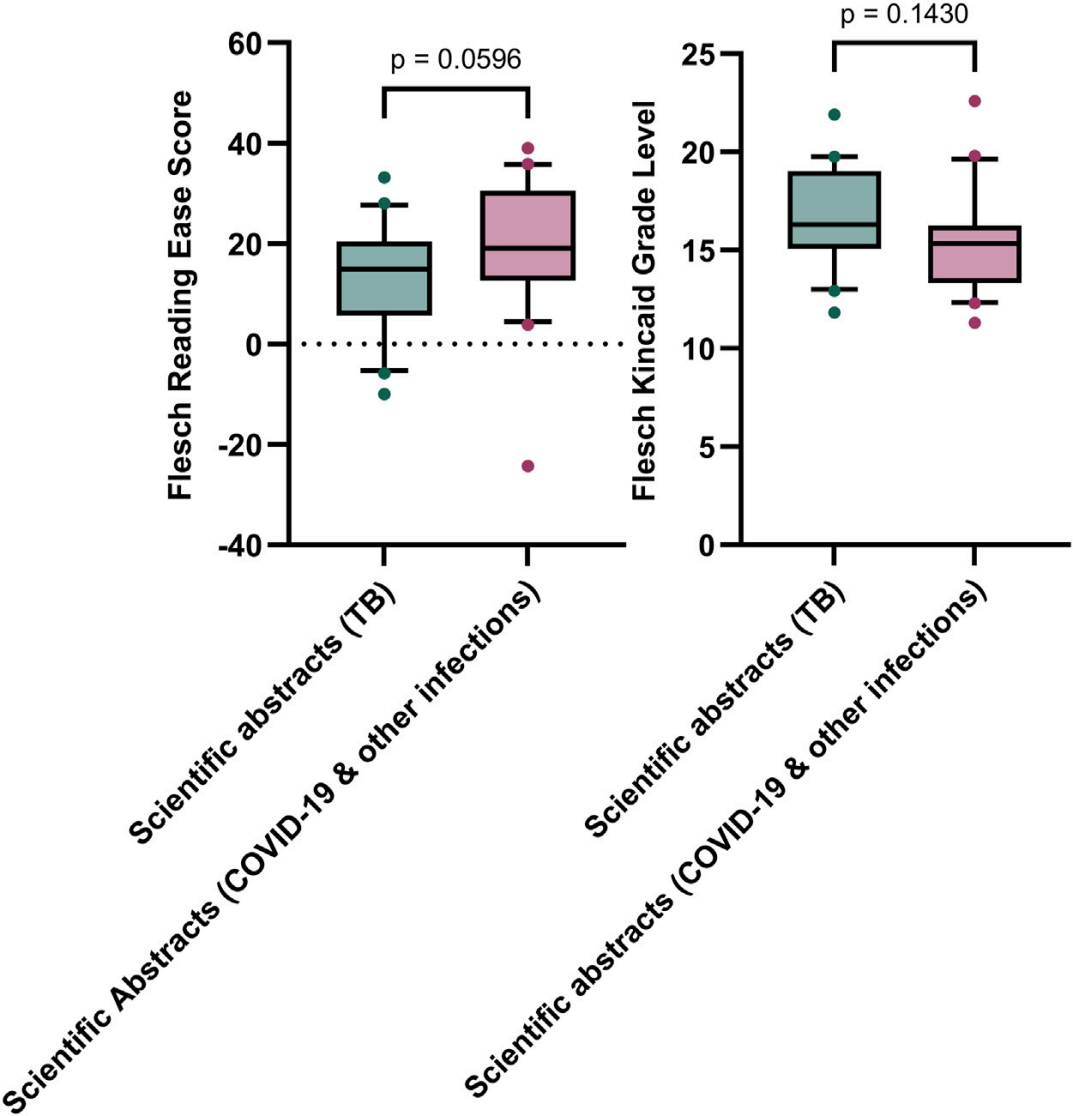
Comparison of the readability (Flesch Reading Ease & Flesch Kincaid Grade Level) of scientific abstracts (TB) (n = 20) and scientific abstracts (non-TB) (n = 20) Box represents 25th and 75th percentile and bar represents the median. Whiskers represent the 10th and 90th percentile and ● represent outliers outside these percentile ranges. Statistical non-significance is shown, calculated using a parametric unpaired t-test for the Flesch Kincaid Grade Level and an unpaired non-parametric Mann-Whitney test. A p-value of <0.05 (5%) was considered as statistically significant.

There was no statistical difference in the Flesch Reading Ease score and the Flesch Kincaid Grade Level, between scientific abstracts (TB) and scientific abstracts (non TB) (Figure 6).

LabTestsOnlineUK yielded a mean FRE score of 51.5 ± 1.2, a mean FKGL score of 10.2 ± 0.5 and text metric scores of 16.7 ± 2.3 and 1.6, for words per sentence and syllables per word, respectively.

## Discussion

**FIGURE 7:**
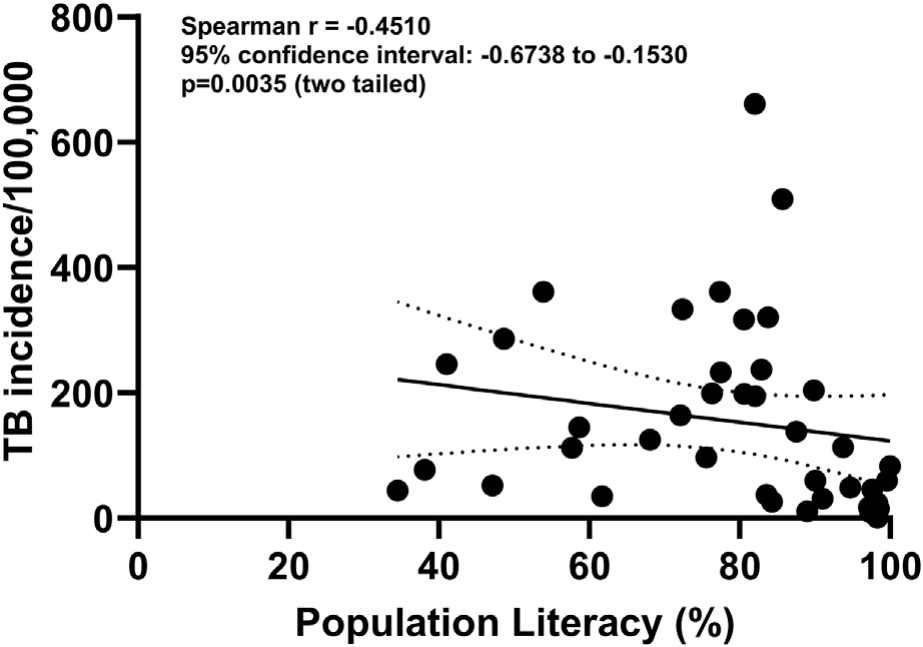
Correlation between population literacy and incidence of TB/100,000 population from 67 countries. Source of data: https://databank.worldbank.org.

Literacy plays an important role in the understanding of tuberculosis [40]. Figure 7 shows the correlation between literacy and the incidence of tuberculosis globally. Whilst this is not a direct cause and effect relationship, it does emphasise the importance of literacy in helping to understand the disease with potential benefits to treatment outcomes and prevention of the spread of infection. Illiteracy is associated with poor adherence and patients’ understanding of the importance of following TB treatment protocols [41]. Therefore, developing resources for TB patients to support treatment literacy of anti-tubculous treatment regimes would be prudent, in order to help patients better understand their antibiotic medications, as well as dosing and treatment durations, in an attempt to maximise treatment adherence.

To our knowledge, the current study is the first to conduct an assessment of the readability of patient-facing, as well as scientific-facing materials which describe multiple aspects of tuberculosis disease, including basic science, epidemiology, symptomology and treatment. In this study, we employed quantitative measurement of words, sentences and syllables, as defined by readability formulae, including Flesch Reading Ease, Flesch Kincaid Grade Level, Gunning Fog and SMOG scores (Table 1), which are commonly described metrics in most readability studies. In the execution of this study, we tried to emulate the journey taken by the patient in trying to find out information on tuberculosis. From examination and comparison of the readability and text metrics results of this study, the overall readability and text metric scores of all combined sources of tuberculosis information did not meet the readability reference targets for either the UK or US.

There is a difference in readability reference targets for the Flesch Reading Ease score and the Flesch Kincaid Grade Level, for US and UK readers, as detailed in Table 4 [16, 42–44]. In the UK, Health Education England (HEE) recommends that patient resources be written at a level suitable for comprehension by an average 11-year-old [16, 42]. In the US, the American Medical Association recommends that all patient-facing material be written at a sixth grade level (11 years old) [44], whilst the US Centers for Disease Control and Prevention (CDC) recommends that patient-facing information is no higher than 8th grade [43, 44].

**TABLE 4:** Comparison of readaility target values in UK and US.

	UK readability target^a^	US readability target^b^
Flesch Reading Ease	≥90	≥60
Flesch Kincaid Grade Level	11 years old (US 6th Grade)	13–14 years old (US 8th Grade)

^a^ Health Education England (HEE) [16, 42].

^b^ Centers for Disease Control and Prevention (CDC) [43, 44].

No information met the UK standard for the Flesch Reading Ease (≥90) and only 2/150 (1.3%) achieved the UK target for the Flesch Kincaid Grade Level (≤6). Twenty three sources of information (23/150; 15.3%) achieved the US reference Flesh Reading Ease score of ≥60 and 20 (20/150; 13.3%) sources reached the US target Flesch Kincaid Grade Level of ≤8. In descending order, TB information from international governments, hospitals and LabTestsOnlineUK were the most readable as defined by the highest mean Flesch Reading Ease scores, of 57.9, 54.1, and 51.5, respectively, whereas scientific abstracts and Cochrane abstracts were the most difficult to read (FRE score = 13.0 and 30.2, respectively). Scientific-facing materials, although not specifically tailored to the general public, are still freely accessible to patients and for this reason, were included within the study analysis. However, as shown in Figure 4, the FKGL and FRE scores for all scientific-facing materials did not meet the required readability targets of either jurisdiction, indicating that the literature would be a difficult read for patients and the public. There was no significant difference between Cochrane Plain Language Summaries and matching Cochrane abstracts, with both having similar difficulty. This finding alone, illustrates that even though plain language summaries are intended to be written in a manner that should be easy to follow and theoretically understandable to the general public, this is not the case.

Clarke and colleagues examined the readability of 17 sources of information relating to the treatment of latent tuberculosis and showed higher readability scores of FKE 63.51 ± 8.81 and FKGL 9.14 ± 8.9, with none of the materials examined meeting HEE recommendations [16]. These authors concluded that current and future LTBI patient education resources would benefit from greater consideration of readability to improve the patient illness experience and support informed treatment decision making. Stossel and colleagues noted that patient education materials are typically written at a college level or higher, making them difficult for the general public to understand and follow [45]. Badarudeen and Sabharwal [43] proposed that by matching the complexity of health education materials to the patients' health literacy levels, it would result in effective communication and perhaps a better understanding of the disease. Similarly, Brennan and colleagues [46] suggested that all patient-facing documents should be prepared and presented to an accessible level in order to meet the needs of the target audience.

Biomedical scientists are important custodians of scientific information relating to tuberculosis for their service user populations, including other healthcare professionals within the TB multidisciplinary (MDT) team, as well as patient service users. Unlike other healthcare professionals, including physiotherapists and dietitians, who have much face-to-face contact with their patient service users, the biomedical scientist’s main mode of communication to this group is through written materials. Examples of these include the description of laboratory tests for tuberculosis, as articulated in the collaborative LabTestsOnlineUK initiative between the Institute of Biomedical Science (IBMS), the Royal College of Pathologists and the Association for Laboratory Medicine (https://labtestsonline.org.uk/). LabTestsOnlineUK has been designed to help the patient to better understand the many clinical laboratory tests that are part of routine care as well as diagnosis and treatment of a broad range of conditions and diseases. The ability to communicate effectively with service users is an important Standard of Proficiency of the biomedical scientist, as detailed by nine descriptors of communication (section 7.1–7.9), as defined by the Health & Care Professions Council (HCPC) [47].

### Resources to Aid Readability

This study has highlighted that nearly all information source materials examined suffered from poor readability. The study also highlighted the need for biomedical scientists to communicate effectively with their service users, as part of their HCPC regulatory Standards of Proficiency. To address this need to improve readability within writing skills and to align more closely with readability targets, we now list some resources and toolkits available to drive enhanced readability in written articles. The NHS Health Research Authority provides valuable resources to help biomedical science authors in writing with enhanced readability (https://www.hra.nhs.uk/planning-and-improving-research/best-practice/writing-plain-language-lay-summary-your-research-findings/). The National Institute for Health and Care Research (NIHR) provides valuable guidance on writing plain language summaries (https://www.nihr.ac.uk/plain-english-summaries). Envision the Patient is a dedicated team within Envision Pharma Group that focuses on bringing the patient into medicine development and who offer a Plain Language Summary Publications Toolkit (https://www.envisionthepatient.com/our-toolkit).

More recently, Attal and colleagues have examined utilising Deep Learning algorithms for automatic adaptation of biomedical abstracts into to plain language versions, through the creation of the Plain Language Adaptation of Biomedical Abstracts dataset [48].

### Study Limitations

Readability describes how easily a piece of text can be read without difficulty by the target audience. Readability considers numerous factors including the complexity of the literature, familiarity with the topic, typography and legibility. Therefore, readability is a crucial component when service users read biomedical information relating to their diagnosis, management and treatment. However, readability does not calculate or assure any level of understanding. For example, if a piece of text narrative has a good readability score, the reader could still find it challenging to understand the topic being discussed.

Readable.com was used within the study due to its advantages, including its accuracy and ability to provide analysis of a range of readability and text parameters [19]. However, one limitation to the tool included its inability to analyse literature in languages other than English, therefore resulting in the study having a lack of inclusivity. This is an important limitation, as the majority of TB and MDR-TB cases occur in regions including Africa, South-East Asia and the Western Pacific, where English is not the first language. For example, countries where the native language is not English, include Bangladesh, China, India, Indonesia, Nigeria, Pakistan, Philippines and South Africa. Consequently, the current study only investigated the readability of TB and MDR-TB health information in countries where English is the native language. Furthermore, this limitation emphasises the need for English-speaking countries to translate health information into other local languages to support endemic populations, particularly in high incidence countries, but also for the movement of peoples due to migration and immigration.

### Identifying Potential Future Work

Acknowledging how health information is delivered, relating to TB and MDR-TB, highlights the manner in which it should be executed in order for it to be better tailored to the needs of the majority of the population. Hence, it is important that the delivery of health information relating to TB and MDR-TB is executed in a manner that is tailored towards the populations’ needs. Thus, providing this information in a range of formats including infographics and leaflets, could help raise awareness about TB and MDR-TB, including what the disease is, how it is diagnosed, and what treatment regimens are available from the appropriate healthcare systems.

Additionally, another area for potential future work is within the scientific community. Scientific papers could potentially include a plain language summary in addition to the scientific paper being published, thereby helping patients and the general public gain the ability to read the information addressed in an easy manner.

## Conclusion

The overall findings of this study conclude that not all freely accessible health information on tuberculosis is delivered in an easily readable format to service users and the general public. In order to meet the basic literacy skills of the general public, readability must be considered by healthcare professionals when producing TB health information and TB patient education materials. Failure to comply with readability standards could possibly lead to health information being misread and therefore potentially misunderstood, potentially resulting in reduced compliance with medical guidance and onwards to poor patient outcomes. To address and combat the increasing incidence rates of TB and MDR-TB, health information should be produced in an easily readable format that service users and the general public find simple and engaging.

## Summary Table

### What Is Known About This Subject

• Tuberculosis (TB) is the world’s leading cause of death due to an infectious agent, with more than 1 billion people dying from the disease over the past two centuries
• Multidrug resistant TB is now emerging as a new public health threat
• TB is a complicated disease with complex biomedical science concepts that service users find difficult to understand. Multiple agencies globally have produced patient-facing information to aid with understanding
• Readability of TB information resources has not be checked to know how well such resouces have been written for service users

### What This Paper Adds

• Readability of TB health information is poor, with Flesch Reading Ease and Fleash Kincaid Grade Level readability scores of 35.6 ± 1.6 (standard error of mean (SEM)) (US Target ≥60; UK Target ≥90) and 12.3 ± 0.3 (US Target ≤8; UK Target ≤6), respectively
• Biomedical scientists are important custodians of scientific information for service users
• When preparing TB information, this should be checked and modified in real time employing readability calculators, to align with health readability targets.

## Concluding Statement

This work represents an advance in biomedical science because it shows that TB information from multiple sources has poorly readability. Effective communication of biomedical science concepts and information relating to TB is vital with service users so that they are able to enhance their health literacy of tuberculosis and clinical outcomes.

## Data Availability

The datasets presented in this article are not readily available because all data is freely available in the public domain. Requests to access the datasets should be directed to je.moore1@ulster.ac.uk.
